# Integrating Case Detection of Visceral Leishmaniasis and Other Febrile Illness with Vector Control in the Post-Elimination Phase in Nepal

**DOI:** 10.4269/ajtmh.18-0307

**Published:** 2018-11-12

**Authors:** Megha Raj Banjara, Murari Lal Das, Chitra Kumar Gurung, Vivek Kumar Singh, Anand Ballabh Joshi, Greg Matlashewski, Axel Kroeger, Piero Olliaro

**Affiliations:** 1 Central Department of Microbiology, Tribhuvan University, Kathmandu, Nepal;; 2 BP Koirala Institute of Health Sciences, Dharan, Nepal;; 3 Public Health and Infectious Disease Research Center, Kathmandu, Nepal;; 4 Department of Microbiology and Immunology, McGill University, Montreal, Canada;; 5 Special Programme for Research and Training in Tropical Diseases (TDR), World Health Organization (WHO), Geneva, Switzerland;; 6 Centre for Medicine and Society/Anthropology, Freiburg University, Freiburg, Germany

## Abstract

Nepal has completed the attack phase of visceral leishmaniasis (VL) elimination and now needs active case detection (ACD) and vector control methods that are suitable to the consolidation and maintenance phases. We evaluated different ACD approaches and vector control methods in Saptari district. We assessed 1) mobile teams deployed in villages with VL cases in 2015 to conduct combined camps for fever and skin lesions to detect VL/PKDL (post–kala-azar dermal leishmaniasis) and other infections; 2) an incentive approach by trained female community health volunteers (FCHVs) in villages with no VL cases in 2015. Both were followed by house-to-house visits. For vector control, four villages were randomly allocated to insecticide impregnation of bednets, insecticide wall painting, indoor residual spraying (IRS), and control. Sandfly density (by CDC light traps, The John W. Hock Company, USA) and mortality (World Health Organization cone bioassay) were assessed in randomly selected households. One VL, three tuberculosis, one leprosy, and one malaria cases were identified among 395 febrile cases attending the camps. Post-camp house-to-house screening involving 7,211 households identified 679 chronic fever and 461 skin lesion cases but no additional VL/PKDL. No VL/PKDL case was found by FCHVs. The point prevalence of chronic fever in camp and FCHV villages was 242 and 2 per 10,000 populations, respectively. Indoor residual spraying and bednet impregnation were effective for 1 month versus 12 months with insecticidal wall paint. Twelve-month sandfly mortality was 23%, 26%, and 80%, respectively, on IRS, bednet impregnation, and insecticide wall painting. In Nepal, fever camps and insecticidal wall paint prove to be alternative, sustainable strategies in the VL post-elimination program.

## Introduction

Visceral leishmaniasis (VL) is a public health problem mostly affecting the poorest of the poor in the tropics.^1^ Until recently, India, Bangladesh, and Nepal contributed 60% of the global VL burden.^2^ Since 2005, under the guidance of the World Health Organization (WHO), these three countries have undertaken a VL elimination program^3^ composed of three successive phases: attack, consolidation, and maintenance. The elimination target is less than 1 case per 10,000 inhabitants at the subdistrict, district, and block level, respectively, in Bangladesh, Nepal, and India initially by 2015.^4^ In 2014, the deadline was further extended to 2017, and Bhutan and Thailand were also included.^5^ Nepal achieved the target in 2014 and Bangladesh in 2016.^6^

The pillars of the attack phase have been active case detection (ACD) combined with treatment at the primary health-care level and vector control through indoor residual spraying (IRS) with insecticides in VL-endemic areas. This has made it possible to identify more cases of VL and post–kala-azar dermal leishmaniasis (PKDL) and treat them earlier, thus, also limiting transmission.^7–9^ These interventions, however, may not be sustainable in the long term, and alternative case identification and vector control strategies are now needed in Nepal to protect the achievements of the attack phase.^10^

Visceral leishmaniasis is now an infrequent cause of fever in Nepal, and a vertical program cannot be maintained. Nevertheless, ACD has proved very useful for identifying VL cases and can be engineered to cover a range of other febrile illness, thus, making integrated disease control and case management possible and sustainable.

For vector control, deploying IRS is cumbersome, expensive, and has limited coverage in space and time, and often is of suboptimal quality in program routine.^11^ Practical alternatives can be explored. Impregnation of existing bednets with slow-release insecticide tablet (KOTAB123, deltamethrin containing slow release insecticide, Bayer, Lyon, France) proved effective in reducing sandfly density and VL disease burden in Bangladesh.^12,13^ Insecticidal paint has been tested against malaria and Chagas disease vectors with encouraging results, and is also effective against pyrethroid-resistant vectors, but has not yet been tested on sandflies.^14,15^

The aim of this study was to investigate and compare intervention strategies combining ACD of VL/PKDL with other febrile illnesses on one hand and strategies for vector control at the community level on the other hand, which could be deployed concomitantly during the post-elimination phases of the VL elimination program in Nepal.

## Materials and Methods

### Study design.

This intervention research study was designed to determine the effectiveness of the integrated ACD and vector control interventions.

For ACD, combined camps for the VL/PKDL cases and other febrile and skin lesion illnesses, including malaria, tuberculosis, and leprosy, were conducted by mobile teams of health workers in villages with VL cases reported in 2015. In parallel, an incentive-based approach for active detection of VL/PKDL along with other febrile cases conducted by female community health volunteers (FCHVs) was tested in villages without VL cases in 2015.

For vector control, we compared interventions consisting of impregnation of bednets with KOTAB123, wall painting of houses with Inesfly paint and IRS using deltamethrin for their effects on sandfly density and mortality in VL-endemic villages.

### Study sites.

The intervention activities were conducted in the VL-endemic Saptari district from June to August 2016 among households of VL-endemic villages. The district has a population of 639,284 living in 121,098 households. The Saptari district has reported decreasing numbers of VL cases detected passively by the routine surveillance system since 2012 to reach an incidence of 0.28 per 10,000 populations in 2014.^16^ This district has one zonal hospital, one district public health office, four primary health-care centers, and 112 health posts. There were 7,211 households with a population of 44,323 in the camp approach area and 9,627 households with a population of 52,277 in the incentive approach area.

Based on the district public health office records for the years 2013–2015, the seven villages with high incidence of VL cases were selected for the fever camp approach, and nine villages with low incidence of VL for the incentive approach–see the following paragraphs.

### Sample size for interventions.

We aimed at assessing, in both study sites, the burden of the four target diseases and calculated the sensitivity of detecting cases through the camp approach (10.7 per 10,000 combined prevalence rates of VL, tuberculosis, malaria, and leprosy) versus incentive approach (9.6 per 10,000 cumulative annual incidence of VL, tuberculosis, malaria, and leprosy)^16^ using as the gold standard the cases detected by house-to-house screening. To get a very high precision of our assessment of the disease burden with a confidence interval close to zero, we decided to include roughly 40,000 inhabitants in each assessment. This would give us sufficient cases to determine the sensitivity of both approaches.

### Case detection interventions.

#### Combined camp approach.

Rapid response teams from the district level were sent to seven villages, which had newly identified VL cases to conduct ACD of VL/PKDL, malaria, tuberculosis, leprosy, and other febrile illnesses, along with focal spraying.

The mobile team consisted of two vector control officers, two doctors, two laboratory technicians, and one health assistant that visited the health post/primary health care center (PHCC) of the area where the new VL cases had been reported to conduct the combined fever camps. Health workers from local health post/PHCC were also included in organizing the camps. Any individual with a history of fever for 2 or more weeks was invited to attend the camp. Awareness activities were conducted before the fever camp with the support of local health functionaries and FCHVs. Camps were held on prescheduled days in previously defined health posts/PHCCs. Camp attendees were screened for VL by asking for chronic fever (> 14 days), palpating spleen enlargement, and conducting the rK39 test. Newly detected cases in the camp were referred to the Sagarmatha Zonal Hospital for further diagnosis, treatment, and clinical and biochemical monitoring for cure. Visceral leishmaniasis patients were reimbursed for travel and food costs and daily wages lost, as per national program guidelines. Cases with skin disease suggestive of PKDL were also tested with the rK39 rapid test for the presence of antibodies against VL.

The serological rK39-negative febrile cases were screened for tuberculosis (sputum samples were diagnosed through GeneXpert, Foundation for Innovative New Diagnostics, USA), leprosy (through clinical examination), and malaria (SD BIOLINE Malaria Ag P.f./P.v. test through SD BIOLINE Standard Diagnostics Korea) and, potentially others. The suspected PKDL cases, if found to be rK39 negative, were tested clinically for leprosy at the campsite and referred to the Lalgadh leprosy hospital for further examination. The detected malaria and tuberculosis cases were referred to the Sagarmatha Zonal Hospital for confirmatory diagnosis and treatment. As per current VL national guidelines, during the fever camp, vector control activities were conducted in the village by the district public health staff with the aim to cover all houses with focal IRS, where there were two or more VL cases identified.

#### Incentive approach.

Female Community Health Volunteers are the village-based female health volunteers in Nepal; they provide referral services to local morbid cases and provide health education to the community. Only those FCHVs who accepted to participate were selected for this activity, which was conducted through the district public health office. All the FCHVs of nine VL-endemic VDCs were identified and a 1-day orientation session was delivered to 49 local health workers and 76 FCHVs by the research team in coordination with the District Public Health Office using training materials and methodology developed and used for the training of VL collaborators by the VL National Programme. Female community health volunteers were trained to identify probable VL/PKDL cases, leprosy, tuberculosis, and malaria and to refer them to the district hospital for confirmation and treatment. Female community health volunteers were informed to provide an incentive Rs. 400 per case as transportation cost after confirmation from the Sagarmatha Zonal Hospital (although FCHVs are volunteers the Nepal government provides Rs. 400 per day per activity as transportation cost). The activities of the FCHVs were recorded and monitored.

#### Blanket household screening and evaluation of interventions.

In the fever camp villages, house-to-house visits covering all households of the intervention area were conducted immediately after the fever camps. In the incentive approach villages, the blanket household screening was conducted 12 months after the training of the FCHVs.

All VL, tuberculosis, malaria, and leprosy cases identified through any of the aforementioned approaches were confirmed and treated according to national guidelines. The patients were reimbursed for travel and food costs and daily wages lost as per program guidelines and norms.

### Vector control interventions.

A randomized controlled trial for vector control was performed to compare bednet impregnation with KOTAB123, wall painting with Inesfly 5AIGRNG^™^ (Inesfly Corp. Spain), IRS with deltamethrin (as per national guidelines), and a no-intervention control group.

Inesfly5AIGRNG contains alphacypermethrin 0.7%, d-allethrin 1.0%, and pyriproxyfen (0.063%). The formulation is vinyl paint with an aqueous base, with the active ingredients residing within CaCO_3_ and resin microcapsules, allowing a gradual release of active ingredients. Microcapsules range from one to several hundred micrometers in size.

Sandfly density was measured in eight villages at baseline to identify four villages with similar sandfly densities. Four villages with no significant differences in the sandfly density were assigned randomly to receive no intervention (control, 92 households) or one of the following three vector control interventions: wall painting (118 households), bednet impregnation (79 households), and IRS (67 households).

After the deployment of the interventions, six households (HHs) from each cluster were selected randomly for entomological analysis, which included sandfly density measurement (baseline and 1, 3, 9, and 12 months after intervention), and WHO cone bioassay to measure sandfly killing on the IRS, wall paint and bednet impregnation (in intervention HHs), and wall (control HHs) at 1, 3, 9, and 12 months after intervention.

#### Sandfly collection and density measurement.

Sandflies were collected during two consecutive nights with two CDC light traps per household by trained personnel under the guidance of an entomologist of the study team, from six randomly selected HHs at 1, 3, 9, and 12 months after the deployment of the vector control activities in each study arm. Sandfly density was calculated as the mean density per CDC light trap per night.

Intervention effect was calculated by the difference-in-difference method using the following formula: (*B* − *A*) − (*D* − *C*), where; *A* = baseline mean sandfly count in the intervention group; *B* = follow-up mean sandfly count in the intervention group; *C* = baseline mean sandfly count in the control group; and *D* = follow-up mean sandfly count in the control group. Negative values indicate the intervention is effective.^17^

#### World Health Organization cone bioassay test.

The cone bioassay test was performed by using the World Health Organization Pesticide Evaluation Scheme (WHOPES) method on 15 randomly selected impregnated nets and 10 control nets, or sprayed and non-sprayed surfaces, or painted walls and control walls, at 1, 3, 9, and 12 months after the intervention. The procedures for the bioassay followed the World Health Organization Special Programme for Research and Training in Tropical Diseases (WHO-TDR) monitoring and evaluation toolkit.^18^

#### Community people’s perceptions of vector control interventions.

The acceptability of vector control interventions was assessed by interviewing the household heads in the study area. The interviews were conducted 1 month after the interventions to collect information on perceived reduction of sandfly and mosquito levels after intervention, and any side effects or inconvenience the intervention might have caused.

### Data management and statistical analysis.

Data were analyzed using IBM SPSS Statistics Version 21, IBM Corporation, USA. Double entry of data was performed for quality assurance. Descriptive statistics were generated. Differences between means were compared by parametric and nonparametric methods depending on the distribution of the variables. Differences between proportions were compared by the chi-squared test.

### Ethical issues.

Study participants signed a consent form. The protocol was reviewed and approved by WHO Ethical Review Committee and by the Nepal Health Research Council.

## Results

### Case detection.

The camps were attended by 398 people (395 with fever for more than 2 weeks and three with skin lesions). The point prevalence of self-reported febrile illness in the seven villages where the camp took place in summer season was 89.12 per 10,000 populations.

Among the 398 people who attended the camp, 275 were further tested: 76 for suspected VL, 97 for tuberculosis, three for leprosy, and 99 for malaria. Of these, one each was positive for VL, leprosy, and malaria; and three were positive for tuberculosis. Immediately following the camp, house-to-house screening of 44,323 people from 7,211 households identified an additional 679 chronic fever and 461 skin lesion cases, none of which was positive for VL, PKDL, tuberculosis, malaria, or leprosy. The camp approach, therefore, identified all of the VL, tuberculosis, malaria, and leprosy in these villages. The overall point prevalence of chronic fever in these villages (between self-reported cases through the fever camp and actively detected cases through blanket household screening) was 242 per 10,000 population (Table 1).

**Table 1:** Findings of house-to-house screening after camp and incentive approach

Particulars	Camp approach	Incentive approach
Number of houses screened (house-to-house)	7,211	9,627
Number of people screened	44,323	52,277
Number of people with fever lasting more than 15 days	679	11
Number of people with skin lesions	461	6
Overall prevalence rate of chronic fever (self-reported and actively detected cases)	242 per 10,000	2 per 10,000
VL, PKDL, leprosy, malaria and tuberculosis identified	6 (VL-1, leprosy-1, malaria-1, TB-3)	0

PKDL = post-kala-azar dermal leishmaniasis; VL = visceral leishmaniasis.

Comparatively, in the incentive approach villages, no case was reported by FCHVs during the year following their training. The blanket screening of 9,627 households was conducted 12 months after FCHVs training (total population 52,277) identified 11 cases of chronic fever and six skin lesions, none of which were confirmed to be either VL, PKDL, tuberculosis, malaria, or leprosy. The overall point prevalence of chronic fever in the incentive approach villages was 2 per 10,000 population (Table 1).

### Vector control interventions.

**Figure 1.**
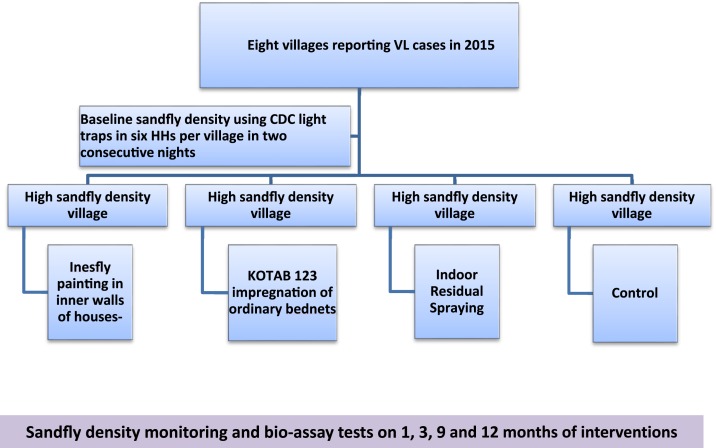
Flowchart of vector control interventions and follow-up. Inesfly paint-Inesfly %AIGRNG TM contains alphacypermethrin 0.7%; d-allethrin 1.0%, and pyriproxyfen (0.063%). KOTAB 123-WHO specified deltamethrin same as used in Permanet. Indoor residual spraying–deltamethrin. This figure appears in color at www.ajtmh.org.

The household and socioeconomic characteristics showing the typical indicators of poverty (high illiteracy rate and agriculture as the only income) were very similar in intervention and control villages. The sandfly density was reduced upto 1 month only by IRS and bed net impregnation, whereas it was reduced for 12 months (last follow-up measurement) by insecticidal wall paint (Figure 1).

Table 2 presents the baseline and post-intervention vector densities at 1, 3, 9, and 12 months. Baseline vector densities in the four villages varied from 2.21 per household per night in the IRS village to the 16.08 in the wall painting village. Table 2 also presents the intervention efficacy as “difference-in-difference” (with 95% confidence intervals) of the sandflies’ densities for 12 months after each intervention application relative to control.

**Table 2:** Effects on sandfly density due to intervention

Month	Sandfly density per household per night				Effects of intervention* (95% CI)			*P*-value comparing with baseline and control		
	IRS	IWP	BNI	Control	IRS	IWP	BNI	IRS	IWP	BNI
0	2.21	16.08	4.96	6.29	–	–	–	–	–	–
1	2.75	7.92	17.38	31.96	−25.13 (−49.23,−1.02)	−33.83 (−60.36,−7.29)	−13.25 (−40.25,13.75)	0.0414	0.0124	0.3370
3	3.36	1.29	4.58	2.42	5.02 (+2.17,+7.86)	−10.92 (−19.30,−2.53)	3.49 (+0.13,+6.84)	0.0005	0.0110	0.0414
9	3.83	0.58	3.75	1.62	5.82 (+3.11,+8.52)	−10.83 (−19.05,−2.60)	3.46 (−0.49, +7.41)	0.0000	0.0110	0.0872
12	23.67	5.29	3.63	6.46	21.29 (+17.70,+24.87)	−10.96 (−20.27,−1.65)	−1.50 (−5.18,+2.18)	0.0000	0.0208	0.4228

IRS = indoor residual spraying; CI = confidence interval; IWP = insecticidal wall paint. Reduction was estimated as the difference-in-difference method. The effect is negative/positive if the sandfly density is decreased/increased after intervention. The effect is zero if there are no changes of sandfly density after intervention.

* Effect of intervention was calculated as: (*B* − *A*) − (*D* − *C*), where, *A* = baseline mean sandfly count for the intervention group; *B* = follow-up mean sandfly count for the intervention group; *C* = baseline mean sandfly count for the control group; *D* = follow-up mean sandfly count for the control group.

**Figure 2.**
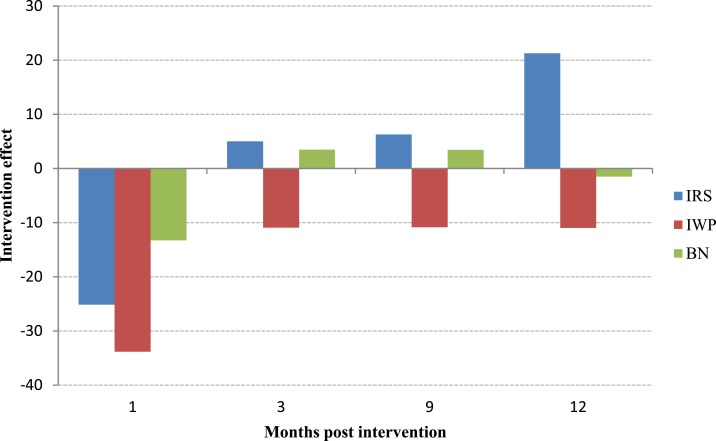
Intervention effect on sandfly density after the application of insecticidal wall paint (IWP), bed net impregnation (BN) with slow-release insecticide, and indoor residual spraying (IRS). Negative values indicate the intervention is effective. This figure appears in color at www.ajtmh.org.

Indoor residual spraying and insecticide-impregnated bednets were effective for upto 1 month after application, respectively, but their efficacy waned thereafter. Insecticidal paint was still effective at the 12-month follow-up measurement (Figure 2).

**Figure 3.**
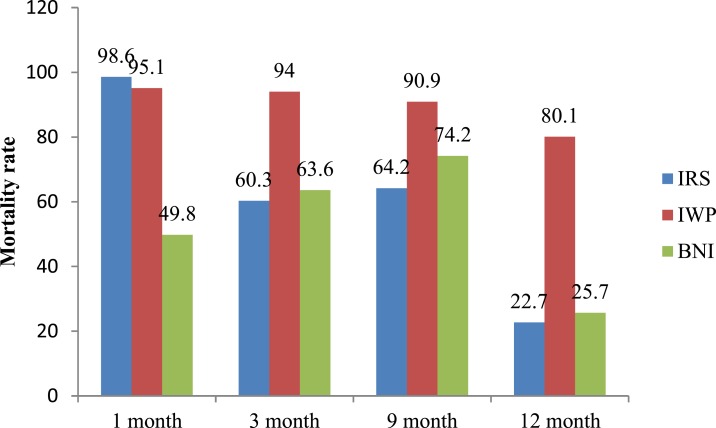
Average Abbot-corrected mortality rate of *P. argentipes* by intervention at follow-up. IRS = indoor residual spraying; IWP = insecticidal wall paint; BNI = bednet impregnation. This figure appears in color at www.ajtmh.org.

The bioassays performed on the treated surfaces showed that the mortality of *Phlebotomus argentipes* sandflies was about 95% at the 1-month follow-up and 80% at the 12-month follow-up on the painted surfaces; 50% and 26%, respectively, for insecticide-treated bednets; and 99% and 23%, respectively, for IRS (Figure 3).

A total of 264 household heads were interviewed for their perceptions and satisfaction about these vector control interventions: 94% perceived a reduction of sandflies after the application of insecticidal paint, 72% after bed net impregnation with insecticide, and 79% after IRS. Overall 10 (seven from insecticide-painted villages, three from bednet impregnation villages, and none from IRS villages) reported mild adverse events (Table 3).

**Table 3:** Perceptions of community people on vector control interventions

Particulars	Village with insecticidal paint (*n* = 118)	Village with slow-release insecticide impregnatio*n* of bednets (*n* = 79)	Village with IRS (*n* = 67)
Perceived reduction of sandfly density after intervention	111 (94.1)	57 (72.2)	53 (79.1)
Adverse events after interventions	7 (5.9)	3 (3.8)	0 (0.0)
Reported adverse events			
Headache	1	0	0
Itching	6	3	0

IRS = indoor residual spraying.

## Discussion

This study was conducted to identify sustainable options to consolidate the achievements of the VL elimination program and eventually break VL transmission in Nepal. This involves coordinated case-finding and vector control activities that can be deployed in the long term and are sustainable. On the one hand, there is the camp approach, which is carried out at one point in time, whereby a team is being dispatched for detecting new cases in highly endemic areas or in areas with a sudden burst in the number of cases. On the other hand, the incentive approach is a continuous activity that can be applied to low-endemicity areas (in our study over a year) and uses the Nepalese system of FCHVs as a community-based “active surveillance” to detect potential VL cases. The study intended to measure the sensitivity of each of these two approaches by conducting a house-to-house survey to see how many cases have been missed. We found that fever camps are effective in identifying active VL, tuberculosis, and malaria cases in the village, thus, achieving the dual objective of potentially reducing VL transmission and providing better care for other causes of febrile illness. We also found that insecticide wall painting provides long-lasting effects and is well accepted by the communities.

This study primarily focused on ACD of VL cases for the maintenance phase of the VL elimination programme. Because all VL-endemic districts of Nepal have achieved the elimination target (incidence of VL below 1 per 10,000 population at the district level), we combined VL and PKDL case detection with tuberculosis, malaria, and leprosy. Among the febrile patients who attended the camp, we detected three cases of tuberculosis, one each of VL, malaria and leprosy, and no case of PKDL. Because no additional such cases were found through the house-to-house screening immediately after the camp, we conclude that this approach is highly effective—thus confirming previous findings—and speaks in favor of broadening the spectrum of febrile illnesses covered by the national communicable diseases control program.^9^ Applying the combined fever camp approach described here would reduce long delays in seeking care for VL diagnosis and treatment and would be more sustainable in the long term.^19^

These observations are also consistent with the overall reduction in VL cases in this endemic district of Nepal. Moreover, this study identified three new cases of tuberculosis. Case identification is one of the major challenges countries are facing in achieving the objectives of the WHO end tuberculosis (TB) strategy.^20^ This approach should, therefore, be considered to complement existing case-finding strategies by the national TB control program in Nepal.

The overall point prevalence of febrile illness in the camp approach villages was 242 per 10,000, of which approximately one-third were self-reported (detected through the camp) and two-thirds were identified through house-to-house screening (which also included more acute fevers). The optimum seasonal periodicity of fever camps still needs to be determined.

The incentive approach implemented through FCHVs did not identify any VL, PKDL, leprosy, malaria, and tuberculosis cases. By contrast, in Bihar, India, the accredited social health activists were able to refer 27% of the VL cases after one training session and 46% after two training sessions.^21,22^ This difference could be due to very low VL caseload in these villages in Nepal (these villages had had no recent VL case) and the need for repeated training of FCHVs.

Although both approaches were applied in the same district, the characteristics of the villages were different. The villages with VL cases had also higher prevalence rates of other tropical diseases and fever in general; those with low endemicity for VL also had much fewer cases of fever and none of the tropical diseases being looked for. It was unexpected to find significantly more febrile cases during the house-to-house screening in camp approach villages than in the incentive approach villages. While the different timing of the two surveys (they were 1 year apart) or increased awareness of fever in the communities which had had recent VL cases could offer potential explanations, such large difference in the point prevalence of febrile cases between the two areas deserves further investigation.

Among the vector control interventions, insecticidal wall paint was effective for upto 12 months after intervention on sandfly density and mortality. By contrast, IRS, even in study conditions, was effective in the first month, but its efficacy waned thereafter both on density and mortality. Bednet impregnation with slow-release insecticide reduced density for the first month and had an effect on mortality for 9 months. The spatial effect of the intervention seems to be crucial to ensure the efficacy of bed net intervention, because bednet impregnation was carried out only in some target houses, and thus, the community effect was lost. Painting of walls with insecticidal paint can therefore become a valuable and sustainable intervention for VL vector control during the maintenance phase of the VL elimination program as its effect lasts longer and is less technically challenging than IRS. It can be combined with other vector control measures (e.g. impregnated bednets), and lends itself to local development for instance through innovative models of social entrepreneurship.

We have now started the economic analysis of the different interventions and their effects, and they can be optimally integrated to produce a durable elimination of VL in Nepal.

## Conclusion

In the maintenance phase of the VL elimination, the combined camp approach offers the dual advantage of being suitable for detecting new cases at an early stage as well as providing a common platform for febrile illnesses at large, hence, being cost-effective and more sustainable. Insecticidal paint can be a valuable alternative to IRS for vector control particularly for the VL post-elimination phase in Nepal.
